# Quercetin Alleviates Osteoarthritis Pain by Inhibiting Vascular Endothelial Growth Factor A Through Regulating cGAS/STING Pathway

**DOI:** 10.1111/jcmm.70992

**Published:** 2025-12-31

**Authors:** Enrui Hu, Yibao Wei, Taiyang Liao, Deren Liu, Zijian Gong, Jun Mao, Peimin Wang, Nongshan Zhang

**Affiliations:** ^1^ Department of Orthopedics Affiliated Hospital of Nanjing University of Chinese Medicine Nanjing Jiangsu Province China; ^2^ Jiangsu Province Hospital of Chinese Medicine Nanjing Jiangsu Province China; ^3^ Key Laboratory for Metabolic Diseases in Chinese Medicine First College of Clinical Medicine, Nanjing University of Chinese Medicine Nanjing Jiangsu Province China

**Keywords:** cGAS/STING, joint pain, knee osteoarthritis, quercetin, VEGFA

## Abstract

Knee osteoarthritis (KOA), a common degenerative joint disease marked by pain, inflammation and cartilage degradation, has been increasingly associated with dysregulated innate immune signalling. Among the implicated molecular pathways, cGAS/STING has emerged as a key modulator in both disease pathogenesis and therapeutic intervention. Quercetin, a naturally derived bioflavonoid with well‐documented antitumour and antioxidant activities, also exerts notable anti‐inflammatory and analgesic effects. This study investigated the mechanistic interaction between quercetin and the cGAS/STING pathway in the context of pain regulation throughout KOA development. Forty‐eight male C57BL/6J mice were randomly allocated into six groups: Sham, KOA, high‐dose quercetin (Que‐H), low‐dose quercetin (Que‐L), STING inhibitor (H‐151) and STING activator (SR‐717). Histological evaluations of entire knee joints were performed using haematoxylin and eosin (H&E) and Safranin O/Fast Green (SO&FG) staining protocols. Serum concentrations of interleukin‐1β (IL‐1β) and tumour necrosis factor‐α (TNF‐α) were measured via ELISA. The viability of dorsal root ganglion (DRG) neurons subjected to PGE2 and quercetin was determined through the CCK‐8 assay. Expression levels of inflammatory and nociceptive markers were assessed using Western blotting, quantitative PCR, immunofluorescence and immunohistochemistry across both in vivo and in vitro models. Quercetin administration led to a statistically significant reduction in peripheral inflammatory and nociceptive markers (*p <* 0.05), diminished pain hypersensitivity and preserved cartilage morphology in KOA mice. These outcomes correlated with the inhibition of cGAS/STING signalling and a concomitant decrease in VEGFA, VEGFR1 and phosphorylated VEGFR1 levels (*p <* 0.05). By inhibiting the cGAS/STING signalling pathway, quercetin mitigates KOA‐related nociception through the downregulation of VEGFA, VEGFR1 and its phosphorylated form.

## 1 Introduction

Osteoarthritis (OA) is one of the most widespread and disabling joint disorders, commonly involving the shoulder, hip and knee and often progressing to chronic pain and functional decline [1]. Knee osteoarthritis (KOA), a predominant form of OA, exerts a considerable impact on healthcare systems, socioeconomic stability and overall quality of life [2]. Defined by the progressive breakdown of articular cartilage, subchondral bone remodelling, ligamentous and capsular disruption, synovial inflammation, infrapatellar fat pad alteration, meniscal degeneration, periarticular muscle wasting, fibrotic changes and osteophyte development, KOA culminates in structural joint damage and impaired function [3, 4]. Pain, the principal clinical manifestation, markedly restricts daily activities and constitutes the primary driver for seeking medical attention [5]. Existing treatment modalities, however, fall short in effectively preventing, managing, or relieving KOA‐related pain [6], highlighting the pressing demand for innovative pharmacological strategies specifically addressing nociceptive and neuropathic pathways.

Quercetin, a naturally occurring flavonoid prevalent in numerous fruits and vegetables [7], exhibits a wide range of pharmacological properties, including cardiovascular support [8], modulation of neural functions [9], antineoplastic activity [10] and immune system regulation [11]. In the setting of KOA, it has been associated with cartilage preservation, inflammatory inhibition, postponement of cellular senescence, suppression of extracellular matrix breakdown, and attenuation of oxidative damage [12, 13]. Owing to its diverse biological functions in counteracting degenerative pathologies [14], quercetin is gaining attention as a viable therapeutic option in the management of KOA [15].

The cyclic guanosine monophosphate (GMP)‐adenosine monophosphate (AMP) synthase (cGAS)/stimulator of interferon genes (STING) signalling pathway constitutes a key regulator of mammalian innate immune and inflammatory pathways [16, 17]. Recent investigations into quercetin‐mediated modulation of this pathway have predominantly centred on its therapeutic potential in ulcerative colitis, oncogenesis and neuroinflammatory conditions [18, 19]. Upon sensing cytosolic DNA—commonly originating from genomic instability—cGAS catalyses the formation of 2′,3′‐cyclic GMP‐AMP (cGAMP), which subsequently activates STING [20]. STING engagement then triggers downstream signalling modules such as NF‐κB, which contributes to articular cartilage deterioration by promoting senescence, apoptosis and extracellular matrix breakdown [21]. Moreover, the pathway has been implicated in regulating the expression of the vascular endothelial growth factor (VEGF) family, a key element in therapeutic approaches to retinal neovascularisation, cardiovascular pathology and ischaemic cerebrovascular disorders [22, 23]. Noteworthy findings in destabilisation of the medial meniscus (DMM) models of KOA revealed that lentiviral‐mediated STING silencing via siRNA mitigated joint degradation and associated pathological alterations [24]. Concurrently, Donnelly CR et al. [25], identified STING as a selective modulator of nociceptive processing through its targeted deletion in peripheral sensory neurons under neuropathic pain conditions. With the expanding mechanistic insights into STING's role in nociception [26], pharmacological targeting of the cGAS/STING pathway is gaining traction as a viable intervention in KOA therapy [27, 28].

The expression levels of VEGF family members exhibit a positive association with the severity of KOA [29]. Intra‐articular VEGF delivery has been implicated in the induction of KOA [30], primarily by triggering inflammatory signalling, promoting synovial fibrosis [31] and accelerating cartilage deterioration through subchondral bone sclerosis [32]. The VEGF family includes five isoforms, among which vascular endothelial growth factor A (VEGFA) remains the most extensively investigated [33]. VEGFA engages two principal receptors—vascular endothelial growth factor receptor 1 (VEGFR1) and VEGFR2 [34], each mediating distinct pathological mechanisms: VEGFR1 is associated with nociceptive pathways [35], whereas VEGFR2 facilitates cartilage matrix breakdown [36]. Accordingly, upregulation of VEGFA and activation of VEGFR1 are increasingly recognised as central drivers of KOA‐related pain, with both targets gaining attention in the development of analgesic interventions for KOA [37, 38].

In this study, a murine KOA model was employed to evaluate the analgesic properties and mechanistic pathways of quercetin. Complementary in vitro assays utilising a prostaglandin E2 (PGE2)‐stimulated cellular system examined quercetin's modulatory effects on VEGFA, VEGFR1, phosphorylated VEGFR1 (p‐VEGFR1) and a range of inflammatory and nociceptive markers. The results indicate that quercetin mitigates KOA‐related pain by suppressing VEGFA expression via downregulation of the cGAS/STING signalling pathway, highlighting its therapeutic promise as a monotherapy for KOA‐associated pain relief.

## 2 Methods and Materials

### 2.1 Reagents

Quercetin (B20527) was obtained from Yuanye (Shanghai, China), and PGE2 (HY‐101952) from MedChemExpress (Shanghai, China). Each compound was independently dissolved in dimethyl sulfoxide (final DMSO concentration < 0.1%) and subsequently diluted in culture medium for in vitro experiments. SR‐717 (T8655) and H‐151 (T5674) were sourced from TargetMol Chemicals (Shanghai, China). Primary antibodies against cGAS (A8335) and CGRP (A24424) were provided by ABclonal Technology (Wuhan, China), whereas those specific to VEGFA (AF5131), VEGFR1 (AF6204), p‐VEGFR1 (AF3204), NGF (DF6061), PGP9.5 (AF5490), TMEM173/STING (DF12090), TNF‐α (AF7014) and IL‐1β (AF5103) were procured from Affinity Biosciences (Cincinnati, OH, USA). The HRP‐conjugated goat anti‐rabbit IgG H&L antibody was also acquired from Affinity Biosciences. The CCK‐8 assay kit (CA1210) and Cell‐Counting Kit‐8 were purchased from Solarbio (Beijing, China). PC‐12 cells (CL‐0480) were obtained from Pricella Biotechnology (Wuhan, China). DMEM, FBS and penicillin–streptomycin solution were acquired from Gibco (Rockville, USA). SYBR Premix Ex Taq II was supplied by Yeasen (Shanghai, China), and qPCR primers were synthesised by Generay Bio (Shanghai, China). ELISA kits for IL‐1β (YFXEM00028) and TNF‐α (YFXEM00031) were obtained from Yifei Xue Bio (Nanjing, China). All reagents were of analytical grade.

### 2.2 Animals

A total of 48 healthy, 8 week‐old male C57BL/6J mice were procured from Qinglongshan Animal Breeding Farm (Nanjing, China). Following a 2 week acclimatisation period under specific pathogen‐free (SPF) conditions—maintaining controlled temperature, humidity and a 12 h light/dark cycle—the animals were randomly allocated into six groups (*n* = 8 per group): Sham, KOA, Que‐H (high‐dose quercetin), Que‐L (low‐dose quercetin), H‐151 (STING inhibitor) and SR‐717 (STING activator). The KOA model was established via DMM as previously outlined [39]. Mice were anaesthetised using 3% sodium pentobarbital (100 mg/kg), and a longitudinal incision was performed to expose the right knee joint. Precise transection of the lateral collateral ligament with ophthalmic scissors was conducted to induce joint destabilisation, followed by meniscal detachment. Model validation was achieved by confirming marked joint laxity with ophthalmic forceps. Surgical wounds were closed using sutures, and postoperative infection was prevented through intraperitoneal administration of penicillin sodium. In the Sham group, only the joint capsule was incised and sutured without manipulation of the ligament or meniscus.

Starting on day 14 following KOA model induction, oral administration of quercetin was initiated in the Que‐H and Que‐L groups at doses of 20 mg/kg/day and 10 mg/kg/day, respectively, in accordance with established protocols [15]. The Sham, KOA and H‐151 groups received matched volumes of 0.9% normal saline. In parallel with quercetin treatment, SR‐717 (30 mg/kg/day) was administered intraperitoneally to the SR‐717 group, while the H‐151 group received H‐151 (7 mg/kg/day) via the same route [40, 41]. At the conclusion of the 4 week intervention period, all animals were euthanised by cervical dislocation. Experimental procedures were approved by the Animal Management Committee and Ethics Committee of Nanjing University of Chinese Medicine (Approval No. 202401A058) and conformed to the guidelines outlined by the National Institutes of Health for the Care and Use of Laboratory Animals. All animal experiments complied with ARRIVE (Animal Research: Reporting of In Vivo Experiments) guidelines.

### 2.3 Histological Analysis

Following euthanasia by cervical dislocation, knee joint cartilage samples were fixed in 4% paraformaldehyde (PFA) for 24 h, followed by decalcification in 10% EDTA for 25 days. Tissues were subsequently paraffin‐embedded and sagittally sectioned at a thickness of 5 μm. Histological staining was performed using haematoxylin and eosin (H&E) and Safranin O/Fast Green kits (Solarbio; G1120 and G1371, China), in accordance with the manufacturers' protocols. Imaging was conducted using an upright light microscope (Eclipse E100, Nikon, Japan) integrated with a digital capture system (DS‐U3, Nikon, Japan). Cartilage degeneration was evaluated using the Osteoarthritis Research Society International (OARSI) scoring criteria [41].

### 2.4 Cell Culture

PC‐12 cells, sourced from Pricella Biotechnology (Wuhan, China), were maintained in complete DMEM supplemented with 10% FBS and 1% penicillin–streptomycin. Culture media were refreshed every 48 h. Incubation was conducted at 37°C under a humidified atmosphere containing 5% CO2.

### 2.5 Primary DRG Neuronal Cell Isolation and Culture

Primary dorsal root ganglion (DRG) neurons were harvested from healthy 8 week‐old male C57BL/6J mice supplied by Qinglongshan Animal Breeding Farm (Nanjing, China). Following cervical dislocation, dorsal skin was rapidly excised, and the L3–L5 vertebrae were isolated and immersed in ice‐cold phosphate‐buffered saline (PBS). Repeated PBS rinses effectively removed residual blood and fur. The spinal cord was accessed via longitudinal incisions along the midline of both ventral and dorsal aspects of the vertebral foramen using ophthalmic scissors. DRGs were then carefully dissected with ophthalmic forceps, transferred to sterile PBS on ice, and subjected to additional cleansing. Subsequent to two further PBS washes, ganglia underwent enzymatic dissociation in 5 mL of 0.2% type I collagenase at 37°C for 1.5 h. After 1 h, digestion was halted by supplementing with complete medium (DMEM containing 10% FBS and 1% penicillin–streptomycin). The tissue was then triturated using a flame‐polished glass dropper, and the resulting suspension was passed through a 70 μm cell strainer to eliminate undigested material. Isolated DRG neurons were plated onto 35 mm poly‐D‐lysine‐coated dishes (1 mg/mL) and cultured in complete medium at 37°C in a 5% CO2 incubator. Medium exchange occurred every 48 h until initiation of downstream applications.

### 2.6 Cell Viability Assay

Cell viability following exposure to quercetin or PGE2 was quantified via the CCK‐8 assay in accordance with the manufacturer's guidelines. PC‐12 cells were plated in 96‐well plates at a density of 1 × 10^5^ cells/well and subsequently treated with graded concentrations of quercetin (0, 2, 4, 8, 16, 32 μM) for 24 h [13], or PGE2 (0, 1, 5, 10, 20, 40 μM) for 5 or 10 min [42]. Posttreatment, 10 μL of CCK‐8 reagent was added to each well, and the plates were incubated at 37°C for 2 h. Absorbance was then recorded at 450 nm using a multifunctional microplate reader (Envision, PerkinElmer, Waltham, MA, USA).

### 2.7 Behavioural Tests for KOA Pain

Behavioural testing was performed on eight mice per group at baseline and on days 14, 28 and 42, within a standardised time window (12:00–17:00). Mechanical sensitivity was quantified via paw withdrawal threshold (PWT), while mechanical allodynia was evaluated by measuring the mechanical withdrawal threshold (MWT) on the plantar surface of the hind paws using a calibrated electronic von Frey device (NC12775, Shanghai Yuyan Instruments Co. Ltd., China) in mice acclimated to Plexiglas chambers (BME404, Institute of Biomedical Engineering, Chinese Academy of Medical Sciences, China). Positive responses were defined by rapid paw withdrawal upon stimulation [43]. Cold withdrawal threshold (CWT) was determined using a temperature‐regulated cold plate (35150–001, Ugo Basile SLR, Italy) maintained at 4°C ± 0.5°C. Behavioural indicators of nociception—including paw shaking, scratching, jumping, lifting, or licking—were systematically recorded [44]. To minimise testing‐related stress and carryover effects, a minimum rest period of 1 h was observed between assessments, and each measurement was conducted in triplicate with 15–20 min intervals.

### 2.8 Immunofluorescence (IF)

IF staining of mouse DRG tissues was conducted in accordance with previously validated protocols [45]. DRG neurons were cultured on glass slides, fixed in 4% PFA for 30 min and permeabilised with 0.5% Triton X‐100 for 15 min. To minimise nonspecific binding, samples were pretreated with 3% goat serum. Primary antibodies targeting cGAS, STING, VEGF, VEGFR1 and p‐VEGFR1 (1:500 dilution) were applied and incubated overnight at 4°C. After thorough rinsing, CoraLite594‐conjugated secondary antibodies were applied for 30 min at 37°C under dark conditions, followed by nuclear staining with DAPI for 5 min. Fluorescence images were subsequently acquired using a DMi8 fluorescence microscope (Leica, Germany).

### 2.9 Immunohistochemistry (IHC)

Cervical dislocation was performed 28 days post‐DMM induction. Lumbar DRGs were harvested, fixed in 4% formaldehyde for 24 h and paraffin‐embedded following decalcification in 10% EDTA and subsequent graded ethanol dehydration. Tissue blocks were sagittally sectioned at 5 μm thickness, dewaxed, rehydrated through an ethanol series and subjected to heat‐induced epitope retrieval. Sections were incubated overnight at 4°C with primary antibodies against cGAS, STING, VEGFA, VEGFR1, p‐VEGFR1, CGRP, PGP9.5 and NGF (1:200 dilution). The following day, secondary antibody incubation was conducted at 37°C for 30 min. After rinsing, chromogenic detection was performed using 3,3′‐diaminobenzidine (DAB). Brightfield images were captured using a Nikon Eclipse E100 microscope equipped with a DS‐U3 imaging system (Nikon, Japan).

### 2.10 Quantitative Real‐Time PCR (qRT‐PCR)

Total RNA was isolated from DRG neurons of C57BL/6J mice using TRIzol reagent in accordance with the manufacturer's protocol. RNA purity and concentration were quantified spectrophotometrically. cDNA synthesis was carried out using the PrimeScript RT kit (Beyotime Biotechnology, Shanghai, China). Gene‐specific primers, designed based on NCBI reference sequences and synthesised by Generay Biotechnology (Shanghai, China), are detailed in (Table 1). qRT‐PCR was conducted on an ABI 7500 Real‐Time PCR System (Applied Biosystems, Life Technologies, USA) using SYBR Premix Ex Taq II. β‐actin served as the internal control, and relative gene expression was determined by the 2^−ΔΔCT^ method.

**TABLE 1:** Real‐time PCR primer sequences.

Name	Description	Primer (5′‐ > 3′)
cGAS	Forward	CAGGAAGGAACCGGACAAGC
	Reverse	CCGACTCCCGTTTCTGCATT
Sting1	Forward	TCTTCTGCCGGACACTTGAG
	Reverse	GAGGTCATGGGGGCATTCAT
Vegfa	Forward	GTGGGACTGGATTCGCCATT
	Reverse	TCCTCCCAACACAAGTCCAC
Flt1	Forward	ACTGTCCTGTGTGGTCAATAAA
	Reverse	CAAGGTTCAGAGTGATGGAGTAA
Uchl1	Forward	CTTGCGTTCTACAGCCCCAT
	Reverse	ACACAGGAGGGAAAGTGCTC
Ngf	Forward	GGGGAGCGCATCGAGTTTTG
	Reverse	GTACGCCGATCAAAAACGCA
Calca	Forward	GTTCTCCCCTTTCCTGGTTGT
	Reverse	TGGGCTGCTTTCCAAGATTGA
Tnf	Forward	CGGGCAGGTCTACTTTGGAG
	Reverse	ACCCTGAGCCATAATCCCCT
Il1b	Forward	AATCTCGCAGCAGCACATCA
	Reverse	GGAAGGTCCACGGGAAAGAC
Actb	Forward	TGAGCTGCGTTTTACACCCT
	Reverse	TTTGGGGGATGTTTGCTCCA

### 2.11 Western Blotting (WB)

After aspirating the culture supernatant from six‐well plates, each well was rinsed with 1 mL of PBS and treated with 300 μL of trypsin. Following a 3 min incubation at 37°C in a 5% CO2 atmosphere, enzymatic activity was quenched by the addition of 1 mL of complete medium. DRG neurons were then gently dissociated using a glass dropper, transferred into 1.5 mL centrifuge tubes and subjected to centrifugation at 1200 rpm for 3 min. The resulting pellet was resuspended in 1.5 mL of PBS and centrifuged again under identical conditions. After discarding the supernatant, 250 μL of RIPA buffer supplemented with 1% PMSF was added for cell lysis. The lysates were mixed thoroughly, incubated on ice for 15 min and centrifuged at 12,000 rpm for 15 min at 4°C. The clarified supernatant was carefully transferred to clean 1.5 mL tubes for downstream analysis. Protein concentration was determined using the BCA Protein Assay Kit (Beyotime, China). Equivalent amounts of protein were resolved via SDS‐PAGE and transferred onto 0.22 μm polyvinylidene difluoride (PVDF) membranes. Membranes were blocked for 1 h and subsequently incubated overnight at 4°C with primary antibodies (1:1000 dilution) targeting VEGFA, VEGFR1, p‐VEGFR1, cGAS, STING, CGRP, NGF, PGP9.5, TNF‐α and IL‐1β. After three 10 min washes with TBST, membranes were incubated with HRP‐conjugated secondary antibodies for 1 h at room temperature on a shaker. Signal detection was performed using the ImageQuant LAS 4000 mini system (Cytiva, USA), and densitometric analysis of immunoreactive bands was conducted using ImageJ software.

### 2.12 Enzyme‐Linked Immunosorbent Assay (ELISA)

Peripheral blood was obtained from the retro‐orbital plexus of C57BL/6J mice after 8 weeks of intervention. Following centrifugation at 3000 rpm for 15 min at 4°C, serum was isolated for downstream analysis. Subsequent to blood sampling, mice were euthanised via cervical dislocation. Quantification of TNF‐α and IL‐1β in serum was performed using ELISA kits, adhering strictly to the manufacturer's protocols.

### 2.13 Statistical Analysis

Statistical analyses and data visualisation were executed using GraphPad Prism 9. Group differences were assessed via one‐way analysis of variance (ANOVA), with subsequent pairwise evaluations conducted using Tukey's post hoc test. Each experiment was independently repeated three times. A threshold of *p <* 0.05 was applied to determine statistical significance.

## 3 Results

### 3.1 Quercetin Reduced Expression Levels of Pain Factors and Inflammatory Factors and Relieved Pain of KOA Mice

**FIGURE 1:**
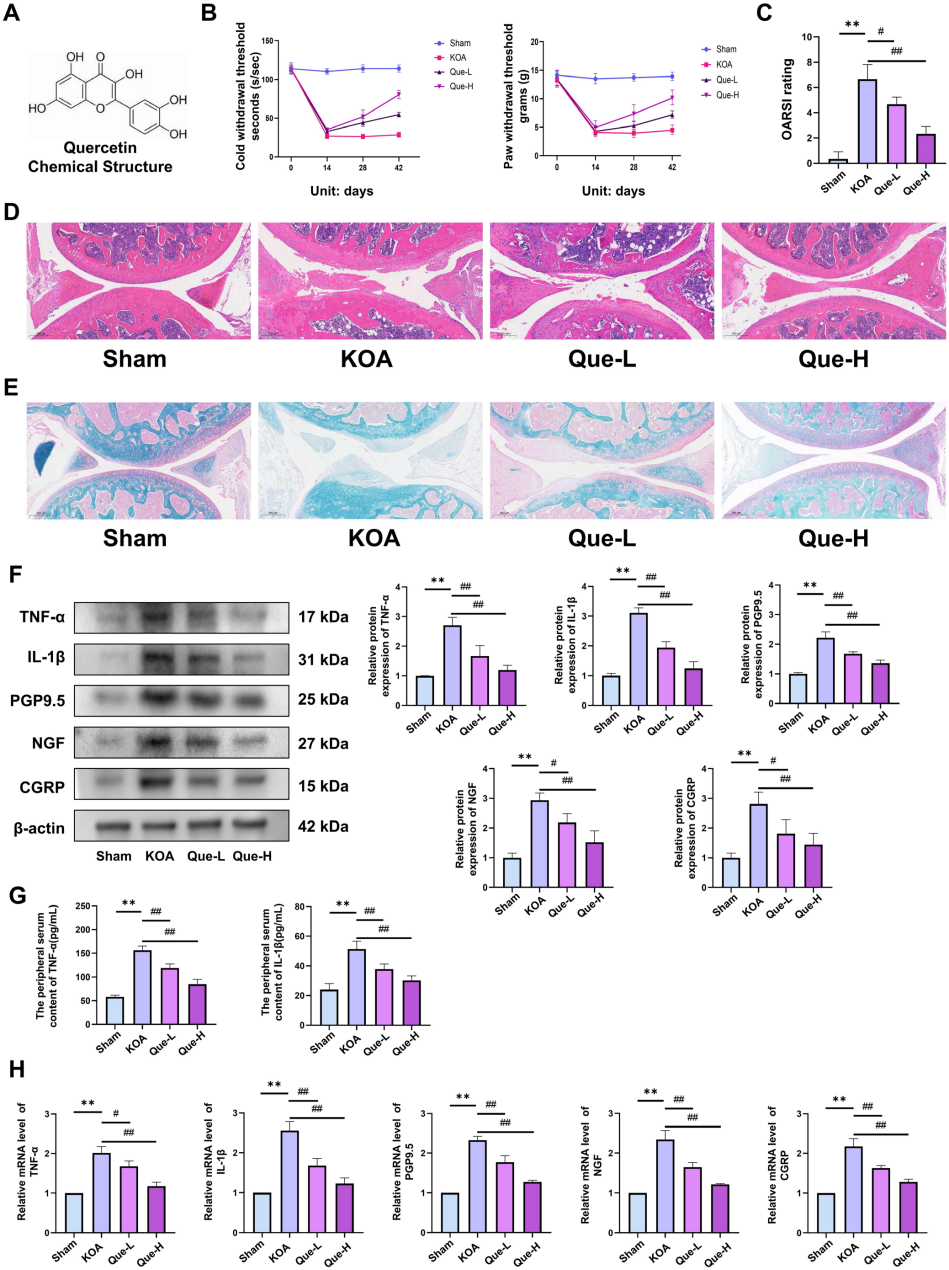
(A) Chemical structure of quercetin. (B) Assessment of cold and mechanical nociception in mice. (C) OARSI scoring results. (D) H&E staining of knee joint sections (×100). (E) Safranin O/Fast Green staining (×100). (F) Western blotting analysis of TNF‐α, IL‐1β, PGP9.5, NGF and CGRP. (G) ELISA quantification of TNF‐α and IL‐1β in serum. (H) qRT‐PCR results for TNF‐α, IL‐1β, PGP9.5, NGF and CGRP. The statistical results are presented as the mean ± standard deviation (mean ± SD, *n* = 3); **, *p <* 0.01 vs. Sham group; *p <* 0.05 vs. other groups; *p <* 0.01 vs. other groups.

Quercetin, a flavonoid extracted from *Forsythia suspensa* , was evaluated for its analgesic efficacy during KOA progression (Figure 1A). Following a 14 day acclimatisation period, DMM surgery was conducted on mice, with quercetin treatment initiated postoperatively. Cold and mechanical nociceptive thresholds were measured on postoperative day 0 and 14, and subsequently on day 14 and 28 after treatment commencement. DMM‐induced KOA mice displayed pronounced nociceptive hypersensitivity by day 14, which persisted through day 28, in contrast to Sham controls (Figure 1B,C). Histological examination using H&E and Safranin O/Fast Green staining revealed substantial pathological alterations in the KOA group, characterised by synovial fibrosis, osteoid deposition and advanced cartilage erosion (Figure 1D,E). In contrast, quercetin administration ameliorated these histopathological features in a dose‐responsive manner. Molecular analyses by WB and qRT‐PCR revealed upregulated expression of CGRP, NGF, PGP9.5, TNF‐α and IL‐1β in DRG tissues of KOA mice, indicative of heightened nociceptive and inflammatory activity (Figure 1F,G). ELISA assays further confirmed elevated serum levels of TNF‐α and IL‐1β. Notably, quercetin treatment resulted in a dose‐dependent suppression of both protein and transcript levels of these inflammatory mediators (Figure 1H). Overall, quercetin markedly reduced KOA‐associated nociception and inflammatory burden (*p <* 0.05), highlighting its therapeutic promise for pain modulation in KOA.

### 3.2 Quercetin Suppressed the Levels of cGAS/STING Pathway

**FIGURE 2:**
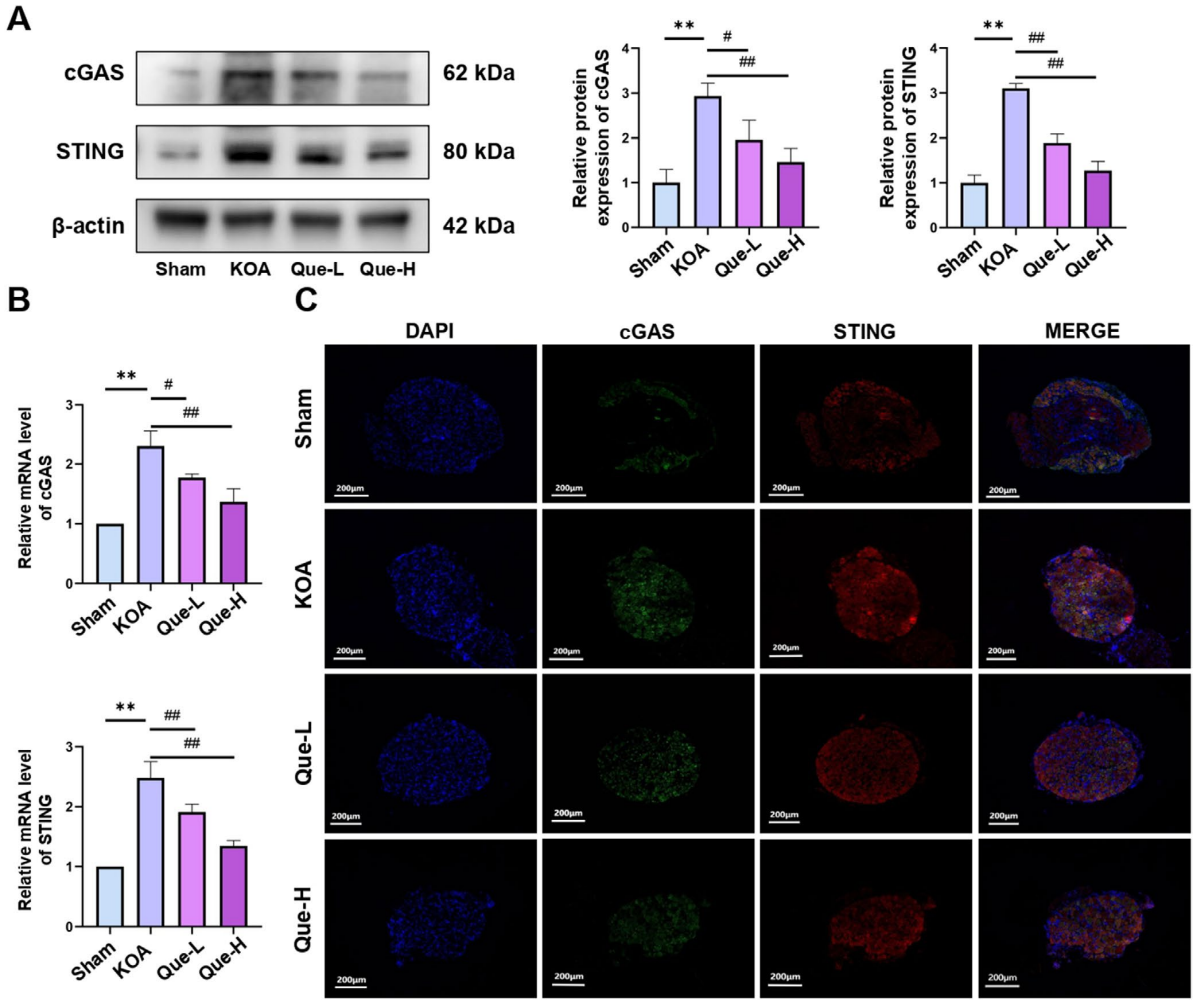
(A) Western blotting analysis of cGAS and STING protein expression. (B) qRT‐PCR analysis of cGAS and STING mRNA expression. (C) Immunofluorescence staining of cGAS and STING in DRG tissues. The statistical results are presented as the mean ± standard deviation (mean ± SD, *n* = 3); **, *p <* 0.01 vs. Sham group; *p <* 0.05 vs. other groups; *p <* 0.01 vs. other groups.

Mechanistic analysis of quercetin's regulatory impact on the cGAS/STING signalling pathway demonstrated significant upregulation of cGAS and STING at both protein and mRNA levels in the KOA group relative to the Sham controls. This upregulation was substantially diminished in the Quercetin‐H and Quercetin‐L treatment cohorts, as confirmed by WB and qRT‐PCR assays (Figure 2A,B). Immunofluorescence staining of DRG tissues corroborated the suppression of cGAS and STING expression following quercetin administration (Figure 2C). Collectively, the data indicate that quercetin mitigates KOA‐related pain through inhibition of the cGAS/STING pathway (*p <* 0.05).

### 3.3 Inhibition of STING Reduced the Expression of VEGFA and VEGFR1 and Suppressed the Phosphorylation of VEGFR1

**FIGURE 3:**
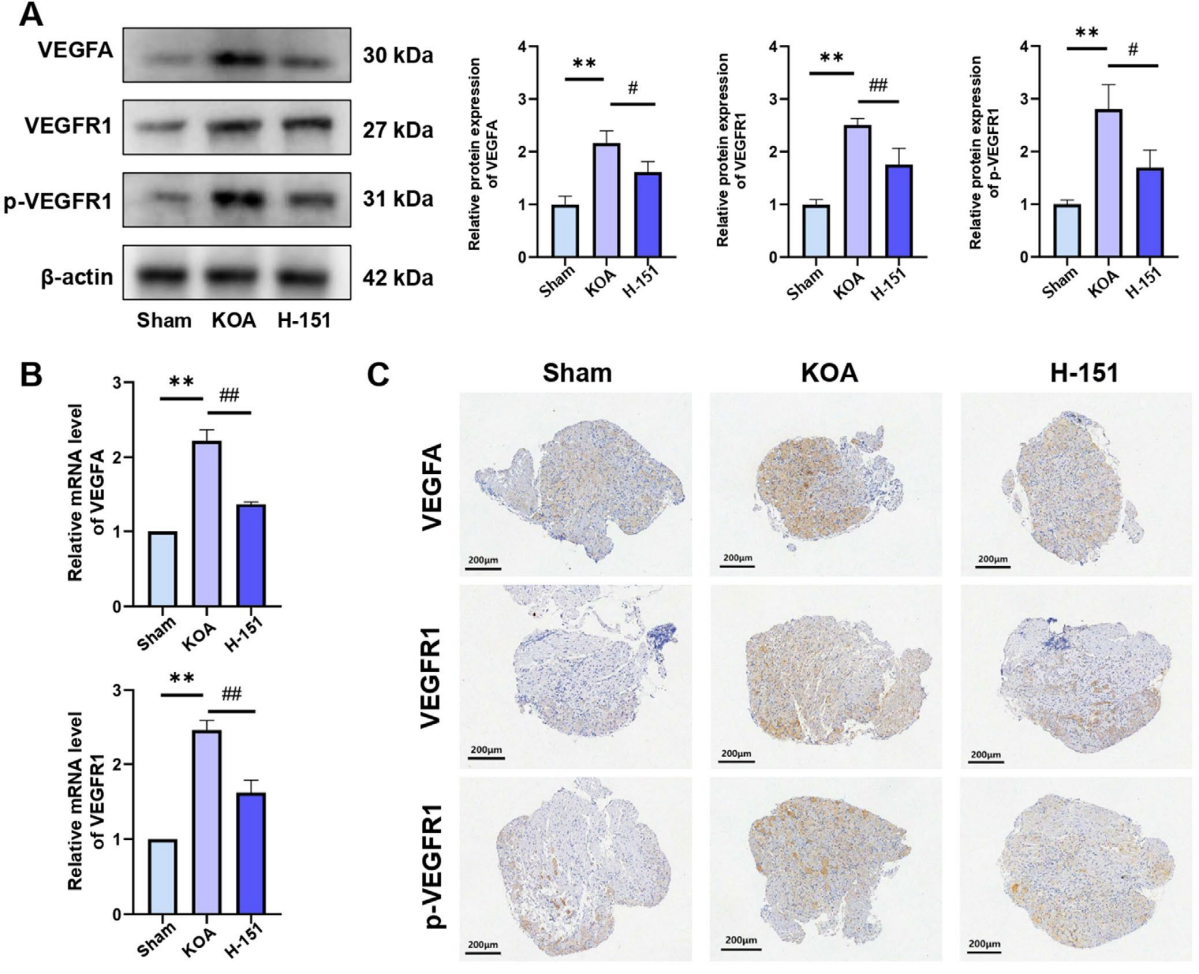
(A) Western blotting analysis of VEGFA, VEGFR1 and p‐VEGFR1. (B) qRT‐PCR analysis of VEGFA and VEGFR1 mRNA expression. (C) Immunohistochemical staining of VEGFA, VEGFR1 and p‐VEGFR1 in DRG tissues. The statistical results are presented as the mean ± standard deviation (mean ± SD, *n* = 3); **, *p <* 0.01 vs. Sham group; *p <* 0.05 vs. other groups; *p <* 0.01 vs. other groups.

To elucidate the functional involvement of STING in KOA‐associated pain, intraperitoneal administration of H‐151—a selective STING inhibitor—was employed to examine the downstream consequences of STING pathway blockade. Western blot analysis indicated a marked reduction in the elevated protein levels of VEGFA, VEGFR1 and p‐VEGFR1 observed in KOA mice following H‐151 treatment (Figure 3A). Correspondingly, qRT‐PCR revealed a significant downregulation of VEGFA and VEGFR1 mRNA expression, which had been previously upregulated in KOA mice (Figure 3B). Immunohistochemistry further substantiated these molecular changes, showing attenuated staining intensity of VEGFA, VEGFR1 and p‐VEGFR1 in DRG tissues of H‐151‐treated mice compared to the KOA group (Figure 3C). Taken together, the data indicate that STING pathway inhibition attenuates KOA‐related pain through suppression of the VEGFA/VEGFR1 signalling pathway (*p <* 0.05).

### 3.4 Quercetin Alleviated Knee Osteoarthritis Pain by Regulating the cGAS/STING Pathway Through Inhibiting the Expression and Action of VEGFA

**FIGURE 4:**
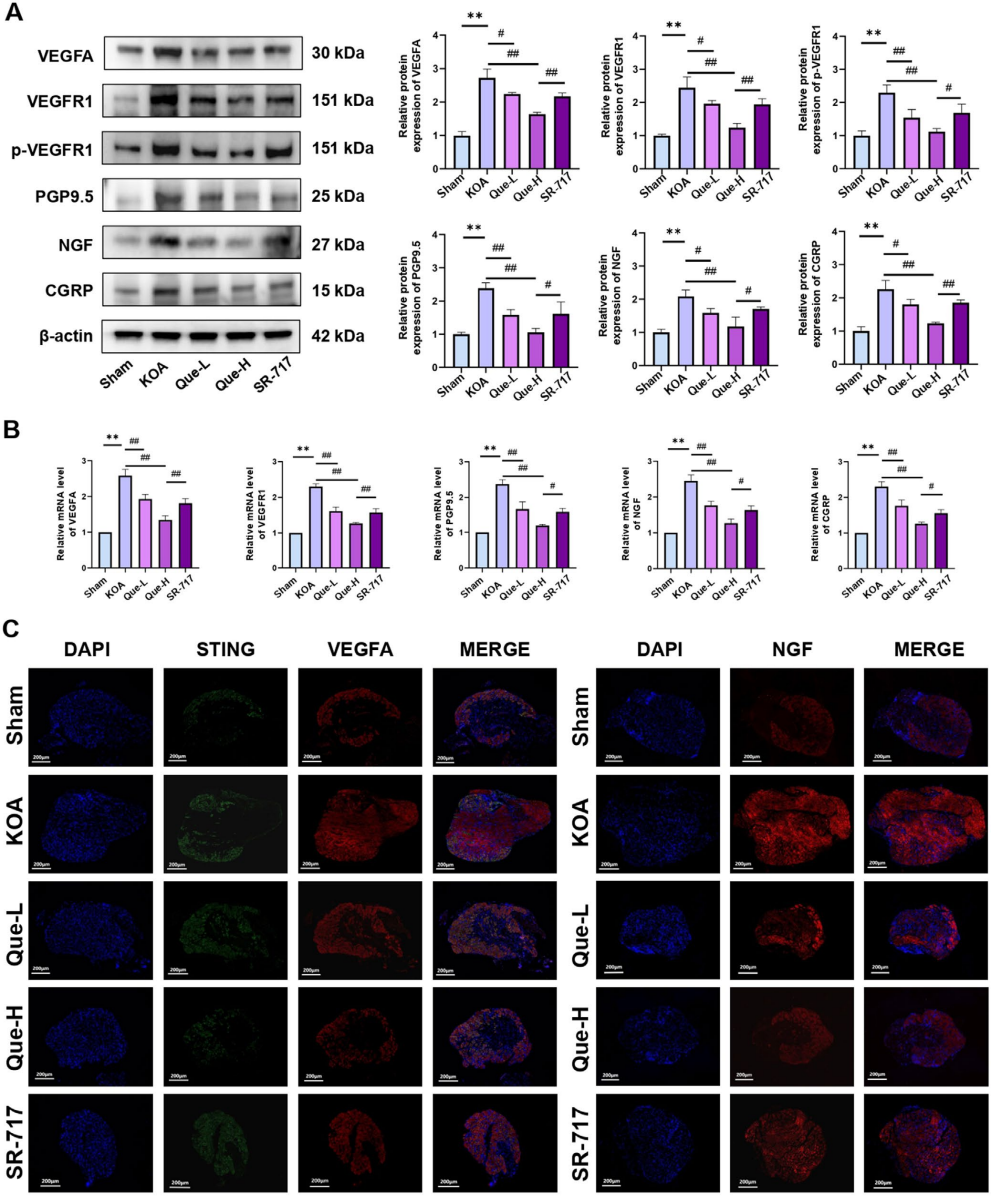
(A) Western blotting analysis of VEGFA, VEGFR1, p‐VEGFR1, PGP9.5, NGF and CGRP. (B) Real‐time PCR analysis of VEGFA, VEGFR1, PGP9.5, NGF and CGRP mRNA expression. (C) Immunofluorescence staining of STING, VEGFA and NGF; the statistical results are presented as the mean ± standard deviation (mean ± SD, *n* = 3); **p < 0.01 vs. Sham group; *p <* 0.05 vs. other groups; *p <* 0.01 vs. other groups.

SR‐717, a cGAMP analog, effectively stabilises STING in its active conformation. Previous reports have identified a direct link between VEGFA, VEGFR1, p‐VEGFR1 and pain manifestations in KOA [46]. Comparative profiling of VEGFA, VEGFR1, p‐VEGFR1, NGF, CGRP and PGP9.5 protein levels, alongside mRNA expression of the corresponding genes, confirmed significant elevation in the KOA group relative to Sham controls. Treatment with quercetin at both low and high doses partially reversed these alterations in a dose‐dependent manner. In contrast, the SR‐717 group exhibited a robust increase in both protein and transcript levels for all analysed markers relative to the Que‐H group (Figure 4A,B). To delineate quercetin's suppressive effects on VEGFA expression and nociceptive markers, protein levels of STING, VEGFA and NGF in DRG tissues were visualised via immunofluorescence. Substantial protein accumulation was evident in the KOA group, while both Que‐L and Que‐H treatments resulted in pronounced attenuation. Conversely, SR‐717 administration intensified the expression of these proteins, likely due to STING activation, which in turn amplified VEGFA signalling and exacerbated pain‐related molecular responses (Figure 4C) (*p <* 0.05).

### 3.5 Quercetin Reduced Expression Levels of Pain Factors and Inflammatory Factors in DRG Neurons

**FIGURE 5:**
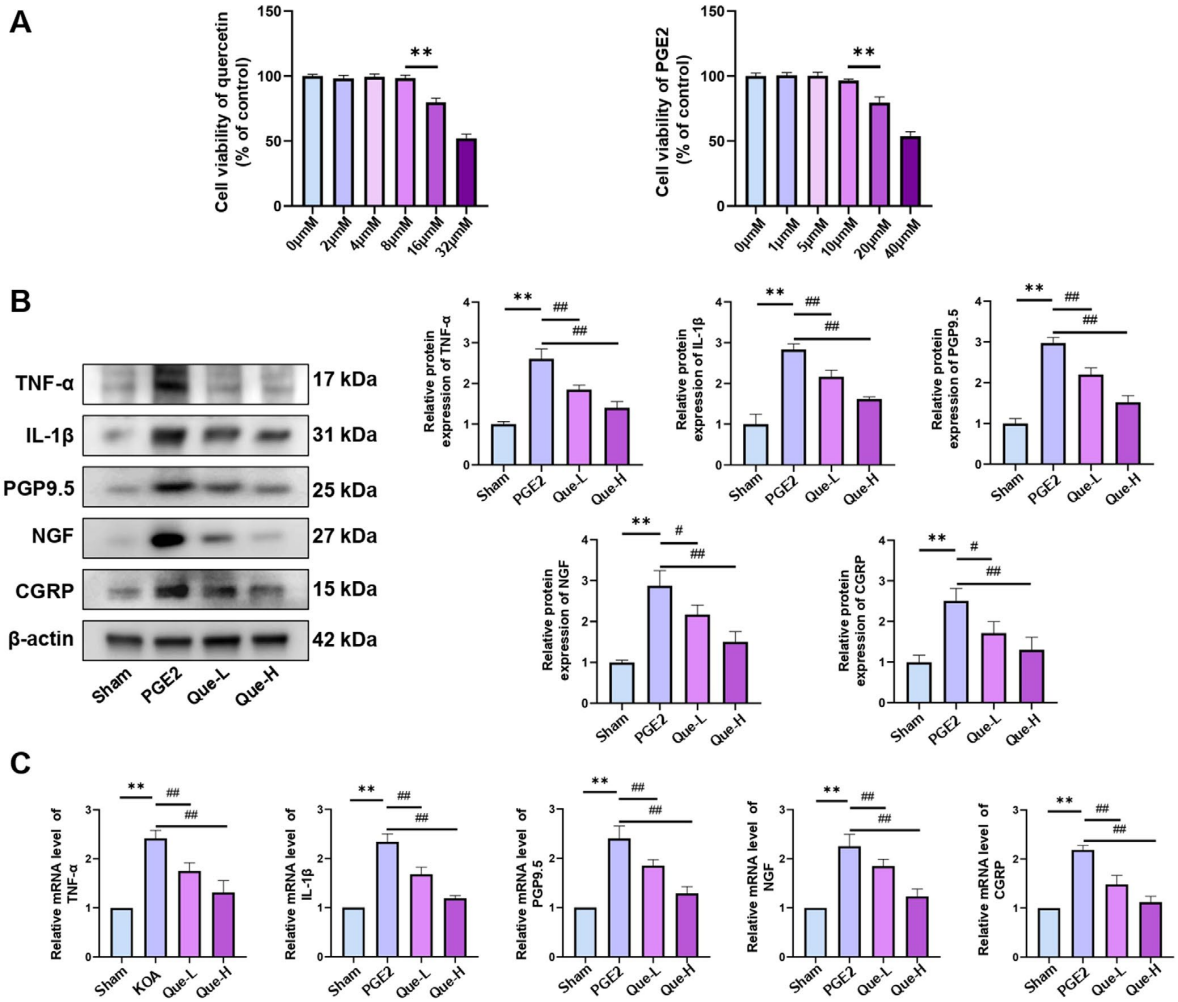
(A) CCK‐8 assay results for quercetin and PGE2. (B) Western blotting analysis of TNF‐α, IL‐1β, PGP9.5, NGF and CGRP. (C) qRT‐PCR results for TNF‐α, IL‐1β, PGP9.5, NGF and CGRP; the statistical results are presented as the mean ± standard deviation (mean ± SD, *n* = 3); **p < 0.01 vs. Sham group; *p <* 0.05 vs. other groups; *p <* 0.01 vs. other groups.

To assess the modulatory impact of quercetin on CGRP, NGF, PGP9.5, TNF‐α and IL‐1β expression in PGE2‐stimulated DRG neurons, cytotoxicity screening was initially conducted. Neuronal viability after treatment with quercetin and PGE2 at concentrations of 0, 2, 4, 8, 16, 32 μmol/L (μM) and 0, 1, 5, 10, 20, 40 μM, respectively, was quantified using the CCK‐8 assay. Quercetin at 2, 4 and 8 μM preserved cell viability, whereas concentrations ≥ 16 μM resulted in significant cytotoxicity. Similarly, PGE2 at 1, 5 and 10 μM did not compromise cell viability, while levels ≥ 20 μM led to a marked reduction. Based on these findings, 10 μM PGE2 was selected to establish a KOA‐like model for subsequent analyses, designated as the PGE2 group (Figure 5A). Quercetin concentrations of 4 μM and 8 μM were adopted as low and high doses, respectively, and corresponding treatment groups were defined as Que‐L and Que‐H. WB and qRT‐PCR data demonstrated a significant upregulation of CGRP, NGF, PGP9.5, TNF‐α and IL‐1β following PGE2 exposure relative to the Sham group, consistent with inflammatory and nociceptive pathway activation. Quercetin administration mitigated this upregulation, with more substantial suppression observed in the Que‐H group compared to Que‐L (Figure 5. B–C) (*p <* 0.05).

### 3.6 Quercetin Suppressed the Expression of cGAS/STING Pathway in DRG Neurons

**FIGURE 6:**
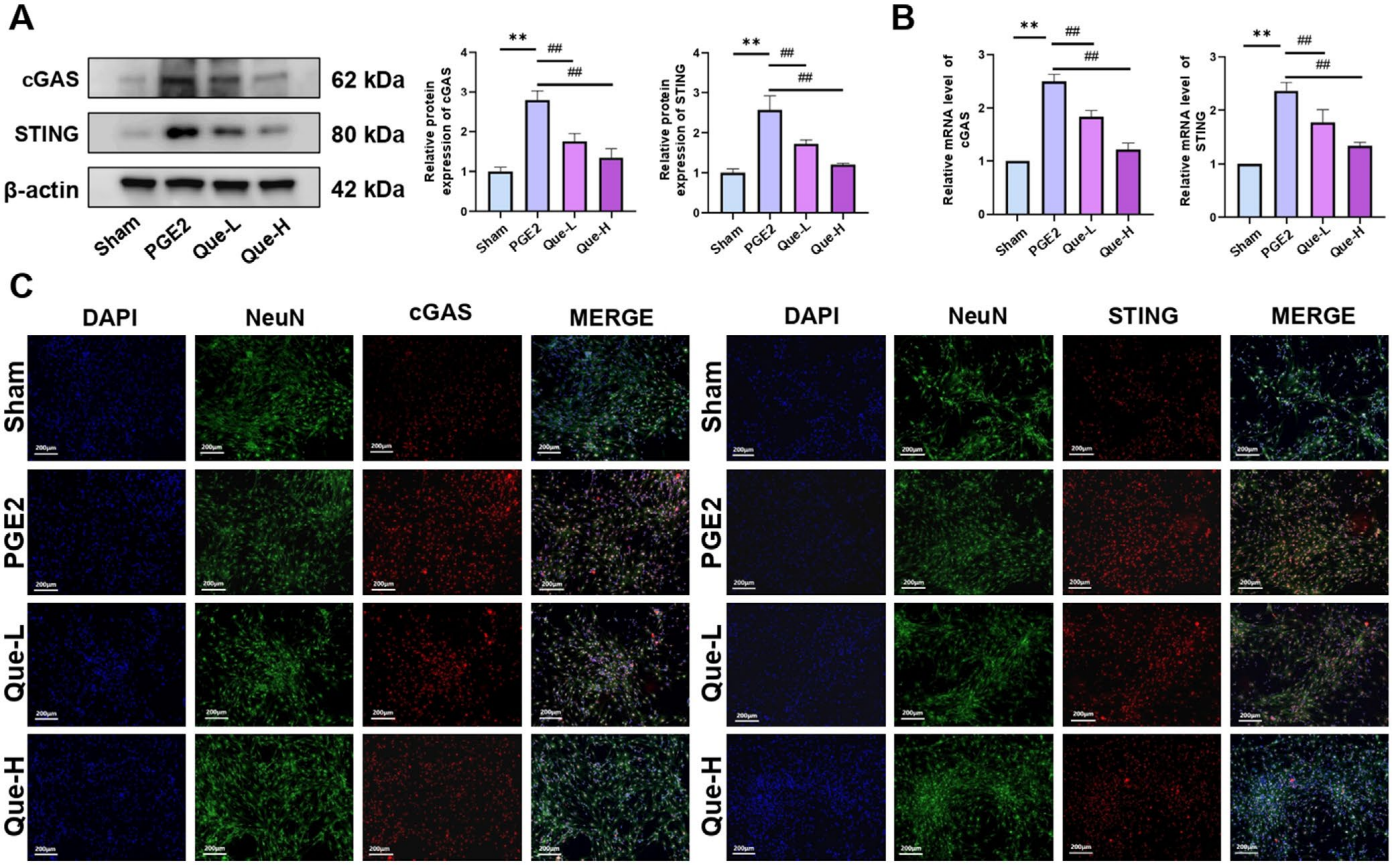
(A) Western blotting analysis of cGAS and STING. (B) qRT‐PCR analysis of cGAS and STING. (C) Immunofluorescence staining of cGAS and STING. The statistical results are presented as the mean ± standard deviation (mean ± SD, *n* = 3); **p < 0.01 vs. Sham group; *p <* 0.05 versus other groups; *p <* 0.01 versus other groups.

To investigate the regulatory mechanism of quercetin on KOA‐related pain, the analysis centred on the cGAS/STING signalling pathway. DRG neurons pre‐exposed to PGE2 were treated with quercetin at concentrations of 4 μM or 8 μM. WB and qRT‐PCR analyses revealed elevated cGAS and STING expression in the PGE2 group relative to Sham, whereas both expression levels were markedly reduced following quercetin administration in the Que‐L and Que‐H groups (Figure 6A,B). Immunofluorescence staining corroborated the transcriptional and translational data, supporting the interpretation that quercetin alleviates KOA‐associated pain through suppression of cGAS/STING pathway activity (Figure 6C) (*p <* 0.05).

### 3.7 Inhibition of cGAS/STING Reduced Expressions of VEGFA, VEGFR1 and Suppressed the Phosphorylation of VEGFR1 in DRG Neurons

**FIGURE 7:**
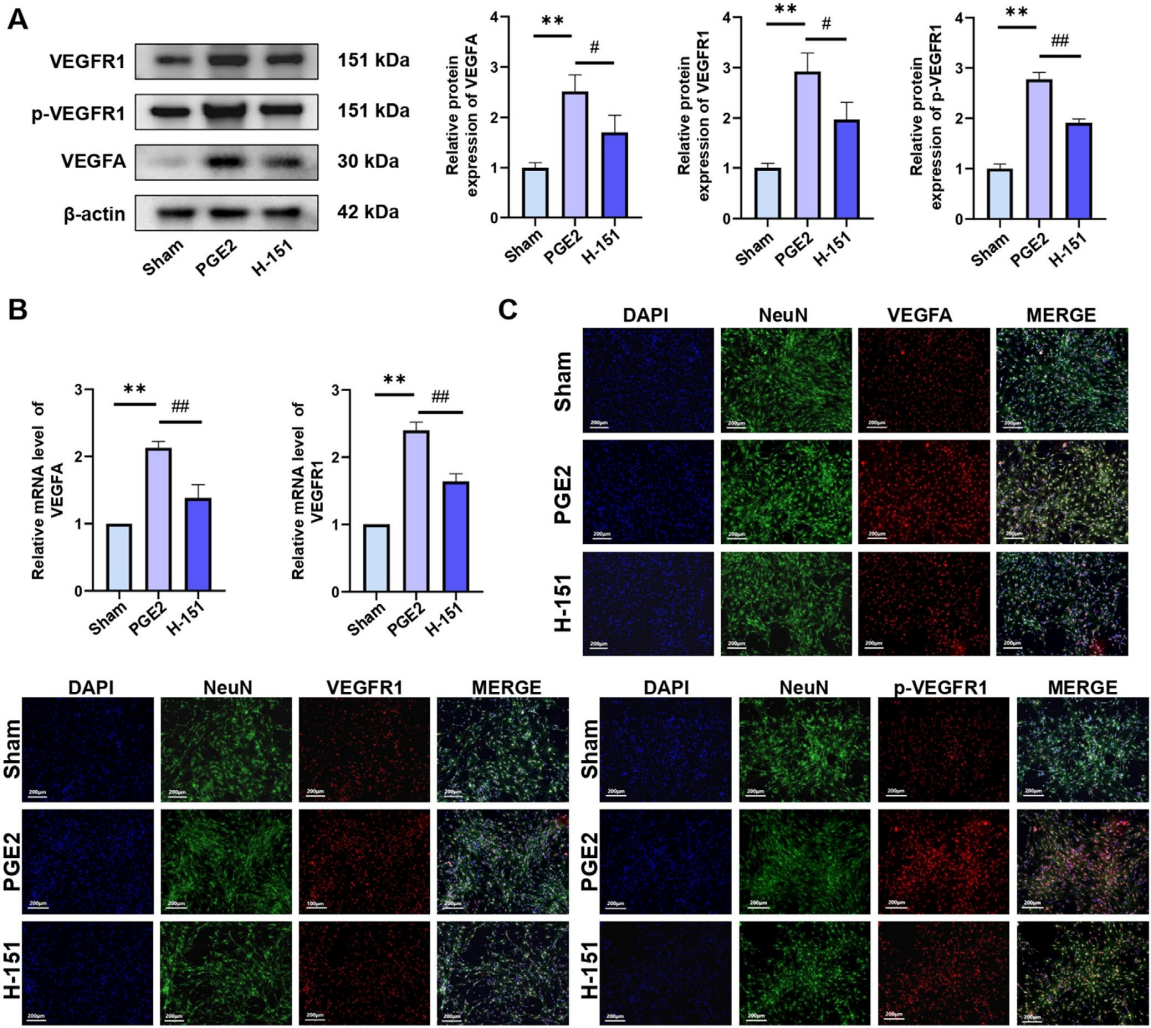
(A) Western blotting analysis of VEGFA, VEGFR1 and p‐VEGFR1. (B) qRT‐PCR analysis of VEGFA and VEGFR1. (C) Immunofluorescence staining of VEGFA, VEGFR1 and p‐VEGFR1; the statistical results are presented as the mean ± standard deviation (mean ± SD, *n* = 3); **p < 0.01 vs. Sham group; *p <* 0.05 vs. other groups; *p <* 0.01 vs. other groups.

To assess the involvement of the cGAS/STING pathway in KOA‐related pain, DRG neurons stimulated with PGE2 were pretreated with H‐151 (0.5 μM) for 2 h before subsequent protein and mRNA analyses [47]. Western blot and qRT‐PCR data revealed pronounced elevation of VEGFA, VEGFR1 and p‐VEGFR1 expression in the PGE2 group relative to the Sham group. In contrast, H‐151 pretreatment substantially reduced the expression levels of these targets compared to the PGE2 group, indicating that pharmacological inhibition of the cGAS/STING pathway attenuates VEGFA signalling and its downstream molecular responses (Figure 7A,B). Immunofluorescence staining corroborated the transcriptional and translational data, supporting the interpretation that H‐151 suppresses the expression of VEGFA, VEGFR1 and p‐VEGFR1 (Figure 7C) (*p <* 0.05).

### 3.8 Quercetin Alleviated Knee Osteoarthritis Pain by Inhibiting the Expression and Activation of VEGFA Factors via Regulating cGAS/STING Pathway in DRG Neurons

**FIGURE 8:**
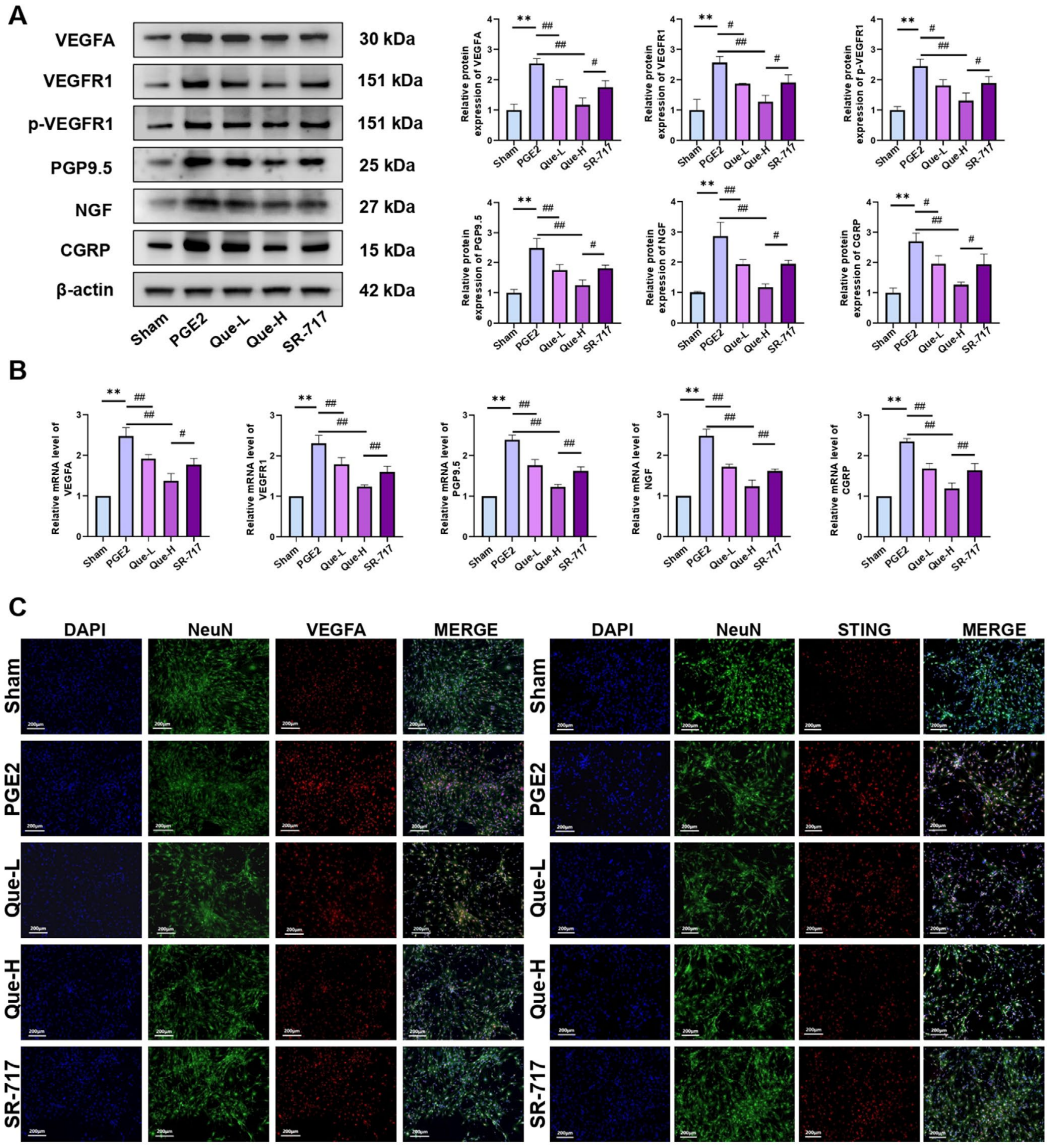
(A) Western blotting analysis of VEGFA, VEGFR1, p‐VEGFR1, PGP9.5, NGF and CGRP. (B) qRT‐PCR results of VEGFA, VEGFR1, PGP9.5, NGF and CGRP. (C) Immunofluorescence staining of STING and VEGFA; the statistical results are presented as the mean ± standard deviation (mean ± SD, *n* = 3); **p < 0.01 vs. Sham group; *p <* 0.05 vs. other groups; *p <* 0.01 vs. other groups.

To validate the role of the cGAS/STING pathway in quercetin‐mediated attenuation of KOA‐associated pain, PGE2‐induced DRG neurons in the SR‐717 group were co‐treated with SR‐717 (3.6 μM) for 2 h prior to analysis [39]. Western blotting revealed elevated protein levels of VEGFA, VEGFR1, p‐VEGFR1, PGP9.5, NGF and CGRP in the PGE2 group relative to Sham, which were markedly reduced following quercetin administration in both Que‐L and Que‐H groups. However, SR‐717 co‐treatment reversed these reductions, resulting in upregulated expression of all evaluated markers compared to the Que‐H group (Figure 8A). Consistent patterns were observed at the transcript level via qRT‐PCR (Figure 8B). Immunofluorescence analysis further demonstrated enhanced expression of STING and VEGFA in the PGE2 group, attenuation in quercetin‐treated cohorts and a reactivation upon SR‐717 exposure (Figure 8C). Collectively, these findings indicate that quercetin alleviates KOA‐related nociception by suppressing the cGAS/STING signalling axis, thereby inhibiting VEGFA expression and VEGFR1 activation (*p <* 0.05).

## 4 Discussion

KOA, a leading subtype of OA, continues to rise in prevalence, largely driven by population aging, increasing obesity rates, and compromised systemic health conditions [3]. Defined by synovial inflammation, cartilage erosion and dysregulated bone remodelling, KOA is principally linked to persistent pain, which remains the predominant impetus for seeking medical care [5]. The inflammatory environment characteristic of KOA promotes the secretion of pro‐inflammatory mediators such as TNF‐α and IL‐1β, which not only amplify nociceptive signalling but also contribute to joint structural degeneration [48]. Current pharmacologic approaches primarily rely on nonsteroidal anti‐inflammatory drugs (NSAIDs) and opioids, which exert analgesic, antipyretic and anti‐inflammatory effects within therapeutic ranges [49]. Nonetheless, extended administration is frequently accompanied by deleterious outcomes, including gastrointestinal toxicity and the risk of opioid dependence [50]. By contrast, herbal therapies have emerged as a promising class of alternatives, offering comparable symptom relief with a more favourable safety profile and presenting a potential trajectory for the advancement of KOA‐targeted analgesics.

Extensive profiling of Sanse powder, a traditional herbal remedy employed in KOA treatment, has identified multiple active constituents via activity‐based component analysis, with quercetin emerging as a principal bioactive compound [51]. As a naturally derived flavonoid present in a wide range of fruits and vegetables, quercetin is characterised by low toxicity and a favourable safety profile. Its anti‐inflammatory, anti‐apoptotic and analgesic activities have been validated across diverse pathological contexts [7, 13, 15]. In KOA, quercetin mitigates disease progression by suppressing extracellular matrix breakdown and attenuating inflammatory cascades at the molecular level [52]. Among the peripheral contributors to KOA pain, DRG neurons represent key modulators of nociceptive hypersensitivity and mechanical allodynia [53]. PGE2, a prominent inflammatory mediator, activates DRG neurons to synthesise and release calcitonin gene‐related peptide (CGRP) [54], a neuropeptide integral to inflammatory pain signalling [55]. By enhancing DRG neuron excitability, PGE2 intensifies peripheral sensitisation and potentiates downstream nociceptive pathways [42]. Concurrently, nerve growth factor (NGF) plays a central role in OA‐related pain mechanisms [56]; its expression is markedly induced under conditions of inflammation and tissue damage, reinforcing its role in persistent pain signalling [57]. Preclinical studies demonstrate that pharmacological blockade of NGF alleviates KOA‐associated pain behaviours in animal models [58]. In parallel, Protein Gene Product 9.5 (PGP9.5), a pan‐neuronal marker present in both sensory and motor nerve fibres, is frequently employed to trace axonal architecture and infer neural engagement in pain pathways [59]. Taken together, CGRP, NGF and PGP9.5 represent viable molecular targets for advancing KOA pain therapeutics.

This study confirmed that quercetin administration effectively mitigated pain‐related behaviours in DMM‐induced KOA animal models and reduced the expression of pain‐associated biomarkers in PGE2‐stimulated cellular systems. Histopathological analysis of knee joint tissues from DMM mice identified hallmark features of KOA, including synovial fibrosis, osteoid deposition, cartilage surface discontinuity and progressive thinning and fissuring of the cartilage layer [31]. Behavioural assays demonstrated a significant decline in both cold and mechanical nociceptive thresholds in the DMM cohort compared to Sham controls. Quercetin treatment, in a dose‐dependent manner, significantly restored nociceptive thresholds, reflecting enhanced pain tolerance.

Immunofluorescence staining revealed elevated levels of nociceptive mediators (CGRP, NGF and PGP9.5) and proinflammatory cytokines (TNF‐α and IL‐1β) in DRG tissues from DMM mice and in PGE2‐induced DRG neuronal models. These molecular alterations were substantially attenuated following quercetin administration. Consistent downregulation of these targets across WB, qRT‐PCR and immunofluorescence assays reinforced the analgesic and anti‐inflammatory potential of quercetin in experimental KOA models.

The cGAS/STING pathway, a newly identified component of the innate immune system, detects cytosolic DNA and triggers downstream proinflammatory signalling pathways [60]. In KOA, joint microenvironmental stress—arising from inflammation or mechanical overload—induces cellular damage, resulting in the release of mitochondrial or nuclear DNA into the cytoplasm [61]. Recognition of this cytosolic DNA by cGAS leads to the synthesis of cGAMP, which subsequently activates STING and initiates inflammatory responses [13, 28, 29, 30, 31, 32, 33, 34, 35, 36, 37, 38, 39, 40, 41, 42, 43, 44, 45, 46, 47, 48, 49, 50, 51, 52, 53, 54, 55, 56, 57, 58, 59, 60, 61, 62]. STING activation enhances VEGF expression in a manner dependent on its signalling activity, culminating in VEGFR engagement [63]. Among VEGF isoforms, VEGFA has been the most comprehensively investigated and is known to modulate pain by selectively activating VEGFR1 on sensory neurons. This interaction regulates transient receptor potential vanilloid 1 (TRPV1) function, promoting Ca2+ influx and initiating ionic currents essential for nociceptive transmission [13, 35, 36, 37, 38, 39, 40, 41, 42, 43, 44, 45, 46, 47, 48, 49, 50, 51, 52, 53, 54, 55, 56, 57, 58, 59, 60, 61, 62, 63, 64]. Mouse models deficient in VEGFR1 signalling within nociceptive (SNS‐Vegfr1^−/−^) or sensory (Adv‐Vegfr1^−/−^) neurons demonstrate significantly reduced pain hypersensitivity [64]. Intra‐articular delivery of anti‐VEGFR1 antibodies alleviates KOA pain, whereas exogenous VEGF administration induces hyperalgesia [13, 30, 31, 32, 33, 34, 35, 36, 37, 38, 39, 40, 41, 42, 43, 44, 45, 46, 47, 48, 49, 50, 51, 52, 53, 54, 55, 56, 57, 58, 59, 60, 61, 62, 63, 64, 65, 66]. Targeted VEGF neutralisation or VEGFR1 inhibition within DRG neurons produces substantial analgesic effects, in contrast to VEGFR2 blockade, which yields limited efficacy [33]. The VEGF/VEGFR1 axis sensitises peripheral nociceptors through modulation of NGF/TrkA signalling, leading to elevated expression of pain mediators and contributing to the pathophysiology of KOA‐associated pain [56].

Following quercetin treatment in DMM‐induced KOA mice and PGE2‐stimulated DRG neurons, convergent evidence from WB, qRT‐PCR, immunohistochemistry and immunofluorescence revealed consistent suppression of VEGFA, VEGFR1, p‐VEGFR1 and core components of the cGAS/STING signalling pathway. The in vivo outcomes closely paralleled in vitro observations, collectively indicating that quercetin inhibited cGAS/STING activation, thereby attenuating VEGFA and VEGFR1 expression and impeding VEGFR1 phosphorylation. This mechanistic interaction implicates the cGAS/STING‐VEGFA axis as a key modulator in quercetin‐mediated analgesia in KOA. To clarify the contribution of cGAS/STING to this effect, pharmacologic modulation was employed: H‐151, a selective STING inhibitor [66], was administered to DMM‐induced mice, while STING activation was induced in quercetin‐treated animals using SR‐717, a synthetic cGAMP analog [13, 40, 41, 42, 43, 44, 45, 46, 47, 48, 49, 50, 51, 52, 53, 54, 55, 56, 57, 58, 59, 60, 61, 62, 63, 64, 65, 66, 67]. Behavioural assays demonstrated that both quercetin and H‐151 significantly elevated mechanical and cold nociceptive thresholds, whereas co‐treatment with SR‐717 reversed quercetin's antinociceptive effects. Corroborative data from IHC, IF, WB and qRT‐PCR analyses confirmed that STING inhibition reduced expression of VEGFA, VEGFR1 and p‐VEGFR1, along with decreased levels of nociceptive mediators (CGRP, NGF and PGP9.5) and proinflammatory cytokines (TNF‐α and IL‐1β) in both DMM‐induced KOA mice and PGE2‐exposed DRG neurons.

Collectively, the results indicate that quercetin mitigates KOA‐associated pain predominantly through inhibition of the cGAS/STING signalling pathway, resulting in reduced VEGFA/VEGFR1 expression and diminished VEGFR1 phosphorylation. Despite the acknowledged limitations—most notably the insufficient characterisation of quercetin's effects on cartilage and synovial tissue—the data delineate a defined analgesic mechanism and provide a basis for subsequent mechanistic and translational studies.

## 5 Conclusion

In summary, quercetin markedly alleviates KOA‐associated pain in both in vivo and in vitro settings, primarily by inhibiting the cGAS/STING axis and downregulating VEGFA, VEGFR1 and p‐VEGFR1 expression. The results indicate that quercetin represents a promising candidate for KOA pain intervention and contributes to the advancement of plant‐based analgesic strategies with favourable safety profiles.

## Author Contributions

**Enrui Hu:** conceptualization (equal), methodology (equal), funding acquisition (equal), data curation (equal), investigation (equal), writing – original draft (equal). **Yibao Wei:** conceptualization (equal), methodology (equal), data curation (equal), investigation (equal), writing – original draft (equal). **Taiyang Liao:** investigation (equal), formal analysis (equal). **Deren Liu:** investigation (equal), formal analysis (equal). **Zijian Gong:** visualization (equal), software (equal). **Jun Mao:** funding acquisition (equal), supervision (equal). **Peimin Wang:** funding acquisition (equal), supervision (equal). **Nongshan Zhang:** validation (equal), project administration (equal), resources (equal), writing – review and editing (equal).

## Funding

This work was supported by the National Natural Science Foundation of China (82274545), Clinical Medical Innovation Center for Knee Osteoarthritis in Jiangsu Province Hospital of Chinese Medicine (Y2023zx05) and Jiangsu Provincial Medical Key Discipline (Laboratory) Cultivation Unit (JSDW202252).

## Conflicts of Interest

The authors declare no conflicts of interest.

## Data Availability

The data that support the findings of this study are available from the corresponding author upon reasonable request.
